# A Checklist for Reproducible Computational Analysis in Clinical Metabolomics Research

**DOI:** 10.3390/metabo12010087

**Published:** 2022-01-17

**Authors:** Xinsong Du, Juan J. Aristizabal-Henao, Timothy J. Garrett, Mathias Brochhausen, William R. Hogan, Dominick J. Lemas

**Affiliations:** 1 Department of Health Outcomes and Biomedical Informatics, College of Medicine, University of Florida, Gainesville, FL 32610, USA; xinsongdu@ufl.edu (X.D.); hoganwr@ufl.edu (W.R.H.); 2 BERG LLC, Framingham, MA 01701, USA; juan.henao@berghealth.com; 3 Department of Pathology, Immunology and Laboratory Medicine, College of Medicine, University of Florida, Gainesville, FL 32610, USA; tgarrett@ufl.edu; 4 Department of Biomedical Informatics, College of Medicine, University of Arkansas for Medical Sciences, Little Rock, AR 72205, USA; mbrochhausen@uams.edu

**Keywords:** clinical research, metabolomics, reproducibility, checklist, reusable data, reproducible workflow

## Abstract

Clinical metabolomics emerged as a novel approach for biomarker discovery with the translational potential to guide next-generation therapeutics and precision health interventions. However, reproducibility in clinical research employing metabolomics data is challenging. Checklists are a helpful tool for promoting reproducible research. Existing checklists that promote reproducible metabolomics research primarily focused on metadata and may not be sufficient to ensure reproducible metabolomics data processing. This paper provides a checklist including actions that need to be taken by researchers to make computational steps reproducible for clinical metabolomics studies. We developed an eight-item checklist that includes criteria related to reusable data sharing and reproducible computational workflow development. We also provided recommended tools and resources to complete each item, as well as a GitHub project template to guide the process. The checklist is concise and easy to follow. Studies that follow this checklist and use recommended resources may facilitate other researchers to reproduce metabolomics results easily and efficiently.

## 1. Introduction

### 1.1. Clinical Metabolomics

Metabolomics is the systematic study of small molecules (i.e., metabolites, which are less than 1500 Daltons and nonpeptides) within cells, biofluids, tissues, or organisms [1,2]. Metabolites represent the downstream output of the genome and the upstream of the environment and have the closest relationship with cell phenotype compared with that of other omics [3]. Clinical metabolomics emerged as a novel approach for biomarker discovery with the translational potential to guide next-generation therapeutics and precision health interventions [4]. For instance, metabolomics was used to identify reasons for side effects and discontinuation of tamoxifen, a medicine to treat breast cancer [5]. Metabolomics imaging was used together with magnetic resonance imaging to identify biomarkers of colon cancer [6]. Metabolomics was also employed to identify biomarkers for multiple other diseases such as diabetes [7] and hepatocellular carcinoma [8], develop drugs such as Enasidenib and Ivosidenib [9], and guide dietary intake [10]. Notably, in the era of coronavirus disease 2019 (COVID-19), metabolomics can be used to develop more advanced diagnostic techniques such as detecting the COVID-19 virus from exhaled air [11]. Metabolomics was also used to identify biomarkers for prognosis and diagnose COVID-19 with bio-fluids [12], as well as predicting the severity level [13]. Although metabolomics is increasingly popular, the literature is flooded with small-scale and preliminary-type studies, many of which also suffer from poor experimental design or statistical validity [4]. Therefore, clinical metabolomics studies with large sample sizes and diverse samples, as well as standardized and robust experimental design, are needed to validate previous findings before results can be reliably applied in real life [2,9,14].

### 1.2. Reproducibility Issue

Reproducibility is challenging for metabolomics research, especially for large-scale ones [15], which is partly due to its high complexity and lack of methodological standardization [15,16,17,18]. For example, Lin et al. recently conducted an interlaboratory metabolomics study regarding reproducibility. They used two labs to process the same sample. The two labs used the same sample preparation protocol but different instrumentation, data processing software, and database, which is a common situation. It turned out that for all metabolites identified by the two labs, only less than half of them were the same [19]. Metabolomics study is complicated, and each step can introduce artifacts into results and hurt reproducibility [19]. However, reproducibility is a must for a novel diagnostic test, vaccine, or treatment to be approved by U.S. Food and Drug Administration (FDA) and used in real life [20,21,22]. Therefore, this article focuses on reproducibility improvement of clinical metabolomics study.

### 1.3. The Checklist

The checklist is a helpful tool to reduce complexity and improve research reproducibility [23]. A checklist is defined as a “list of action items, tasks, or behaviors arranged in a consistent manner, which allows the evaluator to record the presence or absence of the individual listed item” [24]. The checklist was widely used in many situations, such as preventing aircraft accidents and avoiding adverse events in medicine [25]. Relevant checklists were proposed in research fields such as artificial intelligence in dental health research [26] and ecological niche modeling [27].

To improve clinical metabolomics research reproducibility, researchers proposed checklists for research metadata reporting. In 2005, the metabolomics standard initiative (MSI) was formed by leading experts in the metabolomics field [28]. Two years later, several minimum reporting standards (i.e., minimum information checklists [29]) were developed. Summer et al. proposed a minimum reporting standard (MRS) for chemical analysis aspects of metabolomics research, including sample preparation, experimental analysis, quality control, metabolite identification, and data preprocessing [30]. In the same year, Goodacre et al. proposed an MRS specifically for statistical analysis in metabolomics research [31]. Morrison et al. proposed an MRS to report metadata information about biological samples in metabolomics research from an environmental context [32]. Griffin et al. developed an MRS for the description of the biological context of a metabolomics study involving mammalian subjects [33]. Werf et al. also created an MRS for the description of biological information but for metabolomics studies involving microbial or in vitro biological subjects [34]. Fiehn et al. proposed an MRS for metabolomics studies related to plants [35]. Rubtsov et al. developed an MRS for metabolomics research using the NMR data acquisition technique [36]. In 2013, Snyder et al. proposed checklists for metadata reporting for proteomics research and metabolomics research to improve the reproducibility of omics study [37]. In 2020, Long et al. proposed a checklist for metadata reporting of metabolomics studies regarding biomarker discovery [38]. In 2021, Considine et al. argued that the minimum reporting standard developed in 2007 lacked logical flow about data analysis, making it impossible to follow. Then, they created a new checklist and an R markdown template for metadata reporting of data analysis steps in metabolomics research [39]. Recently, Metabolomics standaRds Initiative in Toxicology (MERIT) was launched to develop a minimum reporting standard for clinical metabolomics research in regulatory toxicology [40]. Now it is more than a decade after the minimum reporting standards were proposed in 2007. Nevertheless, several studies found they were poorly followed [29,41,42], which is partly because the information included was overwhelming [39]. A good checklist should be concise [43]. Additionally, existing checklists for reproducibility improvement of clinical metabolomics research were all about metadata reporting. Actions enabling reusable data sharing and reproducible computational workflow development are needed for reproducibility improvement [44,45] but not covered in existing checklists.

### 1.4. Objective

This review firstly covers existing checklists highlighting the metabolomics workflow metadata reporting (Section 2) and then synthesized an eight-item concise checklist, including actions that a researcher can take to facilitate reusable data sharing (Section 3) and reproducible computational workflow development (Section 4).

## 2. Workflow

**Figure 1:**
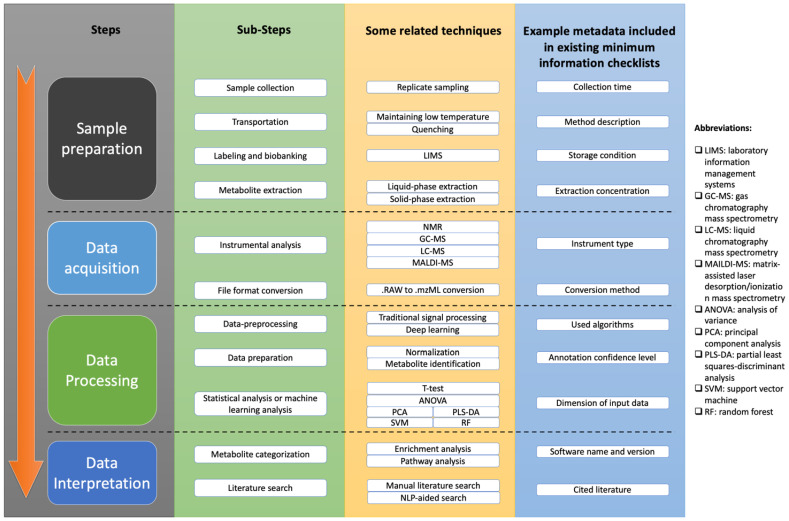
Overview of metabolomics study workflow. Workflow includes steps of sample preparation, data acquisition, data processing, and data interpretation. Each step has multiple substeps, and each substep has several techniques that can be used. Minimum information checklists were proposed to guide metadata reporting for purpose of reproducibility improvement. Some example items included in existing minimum checklists are shown in blue column of figure.

A typical workflow for clinical metabolomics study includes sample preparation, data acquisition, data processing, and data interpretation [46], which is summarized in Figure 1. As we can see, clinical metabolomics studies are very complicated and feature many complex computational workflows with various techniques to generate their results.

### 2.1. Sample Preparation

#### 2.1.1. Overview

Sample preparation includes sample collection, transportation, biobanking and labeling, and metabolite extraction [47]. The requirements and difficulty of sample preparation depend on the sample type and the target disease. Some commonly used samples include blood plasma and serum, urine, saliva, solid tissues, and cultured cells [47]. Notably, even slight variations in this step can affect metabolite stability, influence analytical results, and hurt research reproducibility and credibility [48,49].

#### 2.1.2. Sample Collection

Sample collection is the first and most critical step in clinical metabolomics studies [50], whose quality can determine the quality level of subsequent research [50]. Metadata recommended by existing checklists for reporting include items such as number of sampling replicates, time of collection, species, organ, and cell type [30,35].

#### 2.1.3. Transportation

Collected samples may need to be transported for storage, and stored samples may need to be transported to an analytical laboratory [51]. Maintaining an excellent environmental condition (low temperature) and rapid inhabitation of enzymatic activity (quenching) is essential for preventing quick degradation activity during the process [52]. Sample transportation is recommended to be described as part of the metadata [30].

#### 2.1.4. Biobanking and Labeling

Biobanks store biological samples used for research purposes based on approved protocols [49]. A standardized sample labeling and biobanking approach are vital for research reproducibility [53]. Laboratory information management systems (LIMS) were developed for standardization and reproducibility improvement [54,55]. In terms of clinical metabolomics research example, Rasmussen et al. investigated metabolomics biomarkers of colorectal cancer in blood and used a LIMS named Freezerworks for storage management [56]. Concerning metadata reporting, an existing checklist recommended disclosing information of storage conditions [30].

#### 2.1.5. Metabolite Extraction

Metabolite extraction is the process that separates metabolites from undesired compounds, making the sample and the analyst into a form that is suitable for instrumental analysis [57]. Effective metabolite extraction is required for a successful metabolomics study [58]. The most commonly used extraction approaches are solid-phase extraction (SPE) and liquid-liquid extraction (LLE) [59]. In terms of basic procedures in SPE, a solution is firstly loaded onto a solid phase, such as a cartridge containing the sorbent capable of retaining the target analysis. Then, undesired components are washed away. Finally, desired analytes with another solvent are eluted into a collection tube [60]. Clinical metabolomics studies were conducted with SPE. For instance, Chen et al. used SPE in the process of identifying metabolite biomarkers of lung cancer from exhaled volatile organic compounds [61]. LLE uses water-immiscible solvents to extract interesting analytes from aqueous solutions [62]. Regarding clinical metabolomics research with LLE, Liu et al. used it to investigate the relationship between metabolic alterations and obesity [63]. An existing checklist recommended reporting metadata such as extraction solvent, extraction concentration, extract enrichment, extract cleanup and additional manipulation, and extract storage and transportation [30].

### 2.2. Data Acquisition

#### 2.2.1. Overview

Data acquisition is performed after sample preparation, which consists of instrumental analysis [64] and file format conversion [64].

#### 2.2.2. Instrumental Analysis

Instrumental analysis can be done via nuclear magnetic resonance (NMR) or mass spectrometry (MS). NMR measures the frequency emitted from atoms when an external magnetic field is removed. It can produce a spectrum based on the molecular structure of the compound [65]. MS measures the mass to charge ratio (*m*/*z*) of a molecule by introducing a magnetic field to charged molecules [66]. Various mass spectrometric ion separation/detection approaches are commonly implemented in targeted and nontargeted metabolomics. These are largely driven by the available instrumentation, objectives, hypotheses, and scope of a study. Broadly speaking, this includes high-resolution MS (e.g., using orbitrap or time-of-flight instrumentation) and low-resolution MS (typically using triple-quadrupole mass spectrometers) [67]. High-resolution MS is often used in discovery and nontargeted studies and can provide quantitative and qualitative results. Conversely, triple-quadrupole MS-based methods can only provide nominal-mass spectra but contain a defined list of analytes that can be quantitatively measured with high selectivity and sensitivity. Additionally, “known unknowns” can potentially be characterized retrospectively in high-resolution nontargeted datasets that implement data-dependent or data-independent MS/MS, but not in triple-quadrupole (targeted) studies [68]. MS can also be coupled to orthogonal analyte separation techniques, and thus be further categorized into gas chromatography-mass spectrometry (GC-MS), liquid chromatography-mass spectrometry (LC-MS), and matrix-assisted laser desorption/ionization mass spectrometry (MALDI-MS). In GC-MS, samples are vaporized into the gas phase and separated into various components with a capillary column coated with a stationary phase. GC uses an inert carrier gas such as helium or nitrogen to propel the vaporized samples; then, the mixture’s components are separated. Next, the components or compounds are eluted from the column, and the time of elution is recorded as retention time (RT), which depends on the boiling point (volatility) and polarity. GC-MS is famous for providing high-confidence metabolite annotation [69]; vast GC-MS libraries are publicly available [70,71]. It separates the sample components and introduces them to the MS [72]. Retention time (RT) measures a specific ion or molecule’s time to pass through the column [73]. LC-MS is similar to GC-MS but uses liquid as the mobile phase in the column. Raw GC-MS or LC-MS data includes *m*/*z*, RT, and intensities of peaks [74]. Each peak in the raw data can be an ion, adduct, fragment, or isotope of a metabolite, and one metabolite may be represented by several peaks [75]. MALDI-MS uses a laser energy-absorbing matrix to generate ions from large molecules with minimal fragmentation [76], often used for solid samples such as tissues. Although the above approaches can be used in several fields such as toxicology and proteomics, we focused on the metabolomics field in this review. Some example metadata that need to be reported for this step include the description of the instrument and separation parameters [30].

#### 2.2.3. File Format Conversion

File format conversion is needed when the acquired data files cannot be consumed by the spectral processing software that the researcher has. Popular software for this purpose is ProteoWizard-msConvert [64]. ProteoWizard-msConvert is currently at version 3, it can convert vendor-specific binary metabolomics data files to open-format files, which can be processed with freely available software tools. It provides both graphical user interface (GUI)- and console-based versions. In a recent clinical metabolomics study conducted by Hoegen et al., inborn error of metabolism was analyzed in the study, and ProteoWizard-msConvert was used for metabolomics data file format conversion [77]. Methods used for file format conversion need to be reported as metadata based on the existing checklist [30].

### 2.3. Data Processing

#### 2.3.1. Overview

After data acquisition, data files will be produced in the computer and ready for further processing. Metabolomics data processing includes data preprocessing, data preparation, and statistical analysis [46]. Some popular computational tools (non-commercial) for metabolomics data processing include MZmine [78], XCMS [79], MetaboAnalyst [80], OpenMS [81], and MS-DIAL [82]. MZmine is an open-source, downloadable software written in JAVA. It supports Windows, Linux, and MacOS. It provides a graphical user interface (GUI) as well as console mode. Recently, Teruya et al. used MZmine for LC-MS metabolomics data processing when identifying metabolites related to dementia from whole blood [83]. XCMS has two versions: web-based and downloadable. Researchers can either upload their data to the webserver to analyze or use the R application programming interface (API) locally. Altadill et al. conducted clinical metabolomics research, providing evidence showing that metabolites presented in exosomes-like vesicles could help with explaining the molecular basis of disease progression. Their study used XCMS for LC-MS metabolomics data processing [84]. MetaboAnalyst also provides both web-based and downloadable versions. Liu et al. identified 12 amino acids whose levels are different between Moyamoya disease patients and healthy people; MetaboAnalyst was employed for their metabolomics data analysis [85]. OpenMS is an open-source tool that can process LC-MS metabolomics data. It provides C++ and Python API and supports Windows, Linux, and MacOS. OpenMS was recently used by McCall et al. to detect the metabolic characteristics of fecal pellets from mice that had Chagas disease to identify the impact of Trypanosoma cruzi infection on the gut microbiota [86]. MS-DIAL is open-source software written in C#. It provides both GUI and console versions and supports both Windows and Linux operating systems. MS-DIAL was used by Klont et al. to process LC-MS metabolomics data for the purpose of studying drug use [87]. The output of data processing informs researchers about the intensity of identified metabolites in samples as well as the difference between groups.

#### 2.3.2. Data Preprocessing

Data pre-processing aims to identify peaks representing metabolites in study samples from the raw spectrum. Traditional signal processing techniques are usually involved in the process. Recently, deep learning methods, which is a subfield of artificial intelligence and famous for image processing tasks [88], started to be tested for peak detection and achieved promising performances [89,90,91,92]. According to an existing checklist, detailed methods used in the process of metabolomics data preprocessing should be reported as part of metadata [30]. Notably, the difference of injection order can cause retention time drift and mass to charge drift. To address this issue, sample injection order should also be reported as part of metadata. Signal intensity drift over time is another hurdle of reproducibility, and standard quality control (QC) samples are often used for the correction. Therefore, disclosing both sample injection order and standard QC sample information is critical to ensure reproducible clinical metabolomics research [93]

#### 2.3.3. Data Preparation

Data preparation makes some adjustments, such as normalization to values in the peak table so that the table can be ready for better statistical analysis. Notably, the data normalization method used can dramatically impact the downstream analysis, reporting specific technique used for normalization is important for reproducibility [94]. Some popular normalization techniques for clinical metabolomics studies include median normalization and normalization based on QC samples [95]. Median normalization assumes there is no big change of most of metabolites across samples, and the technique aligns the median signal of all metabolites across samples. QC-based normalization corrects intensities based on QC sample signals, this technique can address the issue of run-order and batch effects [95]. An important step, metabolite identification, is also involved in this process. Metabolite identification can be achieved via matching *m*/*z* value, retention time (RT), or MS/MS spectrum, which may produce identification results with different confidence levels [96]. Based on an existing checklist [30], the confidence level of metabolite identification is an essential part of metadata that needs to be reported for this step; other metadata such as measurements related to unknown metabolites need to be documented as well. Additionally, *m*/*z* drift, RT drift, or other type of signal drift caused by injection-order or batch effects may affect metabolite identification. Fortunately, statistical methods such as non-linear curve fitting can correct the signal if batch information is known [97]. Therefore, reporting information about technical batches as part of metadata is also very important.

#### 2.3.4. Statistical Analysis or Machine Learning Analysis

Statistical analysis is performed after data preparation. The statistical analysis aims to identify differences among groups of samples (e.g., samples from patients before and after treatments) in terms of metabolite volume. Some commonly used statistical analysis techniques include *t*-test and ANOVA [98]. Machine learning analysis can also be used in place of traditional statistical analysis, including unsupervised technique: principal component analysis (PCA); and supervised techniques: partial least squares discriminant analysis (PLS-DA) [99], support vector machine (SVM), and random forests (RF) [100]. Based on existing checklists, metadata such as the dimension of input data and if unsupervised algorithm was used are minimum information that needs to be reported [31,39].

### 2.4. Data Interpretation

#### 2.4.1. Overview

In terms of data interpretation, metabolite categorization, and metabolites literature search are included.

#### 2.4.2. Metabolite Categorization

Literature search aims to identify the relationship between interested metabolites and the research topic. Scientific literature databases such as MEDLINE, Scopus, Google Scholar, PubMed, and Web of Science are the ones that are usually used for searching manually. Additionally, techniques such as natural language processing (NLP) emerge to automate the process [101]. According to an existing checklist for metabolomics metadata reporting, literature cited for interpreting the relationship between metabolites of interest and the research topic must be disclosed [37].

## 3. Reusable Data Sharing

**Figure 2:**
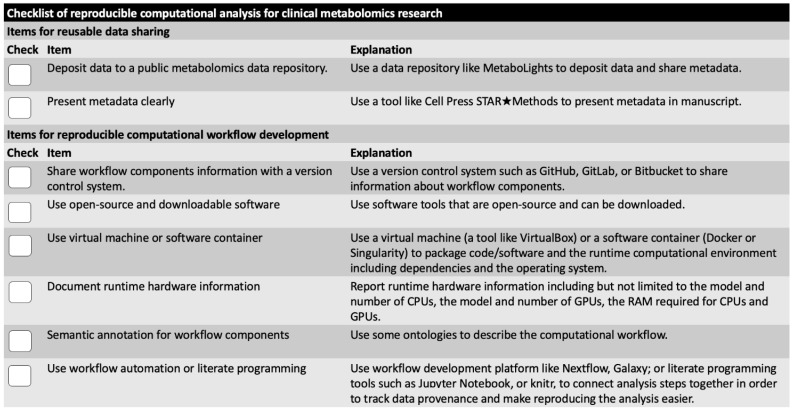
Checklist for computational reproducibility improvement of clinical metabolomics research. Eight items are included, which are categorized to reusable data sharing items and reproducible computational workflow items. All items are about actions that a researcher needs to take for reproducibility improvement. Detailed explanation and example resources are also included on right side of figure.

Two items in the checklist are related to reusable data sharing (Figure 2).

### 3.1. Deposit Data to a Public Metabolomics Data Repository

Making metabolomics data files publicly available is the first step towards reproducible research. Sharing data with the publication is always recommended, but a previous study showed that only a small portion of data from metabolomics research outputs was made publicly available [102]. Many data repositories specifically designed for metabolomics data were developed to facilitate data sharing, such as MetaboLights (https://www.ebi.ac.uk/metabolights/, accessed on 29 November 2021) [103], Metabolomics Workbench (https://www.metabolomicsworkbench.org/, accessed on 29 November 2021) [104], and MassIVE (https://massive.ucsd.edu/ProteoSAFe/static/massive.jsp, accessed on 29 November 2021). Notably, these repositories also adhere to minimum reporting standards [42], which promote data reusability. Numerous recently published clinical metabolomics studies shared data with a public repository. For instance, Neef et al. investigated drug response in colorectal cancer organoids with metabolomics technique and shared their data with MetaboLights (MTBLS2130) [105]. Wu et al. employed a metabolomics technique to identify why the drug Roxadustat as a novel hypoxia-inducible factor stabilizer can protect the kidney from acute ischemic damage [106]. The data were also deposited to MetaboLights with a unique identifier of MTBLS3003.

### 3.2. Present Metadata Clearly

To embrace reproducible research, in addition to depositing data and metadata online, several journals such as Nature and Cell started requiring authors to submit and report experimental metadata in the manuscript. Presenting metadata clearly in the manuscript is another step towards reproducible research. A clear presentation means the presented information can be understood immediately, and readers can absorb and apply it efficiently and correctly [107]. STAR Methods from Cell Press is an excellent tool for clear scientific metadata presentation [108]. STAR (Structured, Transparent, Accessible Reporting) Methods is a template introduced in the fall of 2016. It aims to reflect the changing needs of the scientific community for increased clarity and transparency in reporting of approaches to foster rigor and reproducibility in research. In 2019, STAR Methods was expanded to an open-access journal named STAR Protocols. It is recommended to format the metadata and detailed method following STAR Methods and submit the protocol to STAR Protocols. By doing so, the method and protocol will be improved collaboratively by authors, reviewers, and editors, and reproducibility will be significantly improved [109]. Notably, STAR Methods was used by clinical metabolomics studies. For instance, Li et al. conducted research to evaluate the response to vaccination in humans, including metabolomics signatures [110]. Their study protocol was described in their supplementary material following STAR Methods formats. STAR Protocols also started to publish metabolomics protocols such as metabolite detection in human embryonic stem cells [111]. The protocol was employed for related research [112,113,114] and can improve the reproducibility of research. Therefore, to improve reproducibility, it is recommended to present metadata clearly by writing a STAR Protocol along with the clinical metabolomics research.

## 4. Reproducible Computational Workflow Development

Six items are included in the checklist are for actions regarding reproducible computational workflow development (Figure 2).

### 4.1. Share Workflow Information with a Version Control System

It is recommended to share information of the computational workflow with version control systems, which is a popular way for project management [115]. Additionally, the order of using or executing the computational workflow components should also be documented [45]. Commonly used platforms for computational resource sharing include GitHub (https://github.com/, accessed on 29 November 2021), Bitbucket (https://bitbucket.org/product, accessed on 29 November 2021), and GitLab (https://about.gitlab.com/, accessed on 29 November 2021). In terms of an example clinical metabolomics research, Alvarez–Mulett et al. investigated metabolomic signatures defining clinical outcomes in severe COVID-19 patients and shared workflow information such as code on GitHub [116]. We also developed a GitHub template for this purpose: https://github.com/lemaslab/reproducible_metabolomics_study_checklist, accessed on 29 November 2021.

### 4.2. Use Open-Source and Downloadable Software

To promote reproducible research, using open-source and downloadable software is recommended. Nonavailability of code is a severe reproducibility impediment and may prevent researchers from analyzing the reason for failing to reproduce the original research [117,118]. A web-based (non-downloadable) software may hurt reproducibility by precluding users from accessing older versions [119]. As mentioned in the introduction, software tools such as MZmine and MS-DIAL are open-source and downloadable and were used in clinical metabolomics studies. Additionally, if any self-written code is included, making it open-source and downloadable is also recommended.

### 4.3. Use Virtual Machine or Software Container

Studies indicated workflow component differences hurt reproducibility [45,120]. Workflow components differences include software, code, operating system, and computer hardware [45,120]. Notably, even if related information was reported as part of metadata, it can be cumbersome to obtain previous software versions, and the specific operating system used by the original research might not be at one’s disposal [121]. Fortunately, software containerization and virtual machine (VM) enable researchers in the different labs to run software tools and code with the same computational environment. A software container is a lightweight, standalone, and executable package of software that includes the software/code, its dependencies, and settings. Software containers encapsulate operating system (OS) components, scripts, code, and data into a single package that can be shared with others. Containerized software or code can be run with dependencies installed within the container, which is isolated from packages or dependencies already installed in the host system. Nowadays, both console-based software and software with graphical user interface (GUI) can be containerized [122,123], and the software container supports both Linux- and Windows-based applications [124]. Some commonly used software containerization tools are Docker and Singularity [125,126], but Singularity has better support towards high-performance computing [127]. However, software containers interface directly with the host OS, reducing flexibility since software containers are specific to a given type of OS. A VM does not rely on the OS of the host machine, and thus is more flexible. However, a VM is preferred over a software container when a software whose running environment is different from the host OS [125]. VM uses a hypervisor that sits between physical hardware and virtualized environments to enable multiple virtual OS to be created from the same hardware. Nevertheless, VM requires considerably more computational resources than software containers; it needs more time for initialization and takes up more storage. VirtualBox [128] is a popular and freely available hypervisor.

### 4.4. Document Runtime Hardware Information

Hardware differences can still produce different results when running the same code to process the same data [45]. Running code with software containerization or VM does not fully insulate the environment from the underlying hardware. For example, researchers may find their graphical user interface-accelerated code fails to produce the same results on other machines due to hardware differences [45]. Therefore, it is also recommended to record hardware information including but not limited to the model and number of central processing units (CPUs), the model and number of graphics processing units (GPUs), the amount of random-access memory (RAM) required for CPUs and GPUs.

### 4.5. Semantic Annotation for Workflow Components

Workflow decay is another factor that hurts reproducibility [129]. Workflow decay means the workflow is not well-maintained and is outdated when a second researcher wants to reproduce a previous study that used the workflow [130]. Notably, reporting the metadata information of computational workflow used for the research cannot ensure the workflow will not be outdated or unavailable at the time a second researcher is trying to reproduce the study. Fortunately, semantic annotation of workflow components with controlled vocabulary underlying an ontology can benefit workflow preservation and protect the workflow from decay [130]. Even if the workflow is outdated, such annotations may enable other researchers to create a similar workflow that may regenerate the original results [131]. Table 1 illustrates some ontologies that can provide semantic annotation for computational workflow. Specifically, Research Object Ontology annotates research objects associated with a workflow such as the author, hypothesis, and conclusion. Workflow Description Ontology describes workflow specifications such as input, output, and parameters of a process. Workflow Provenance Ontology describes the provenance traces obtained by executing workflows. Research Object Evolution Ontology tracks the changes of workflow objects [130]. OntoSoft [132], OntoSoft-VFF [133], and Software Description Ontology [134] capture scientific software metadata. Description of a Software Project (DOAP) ontology [132] can be used to annotate things like issues, bug tracking, and wiki discussions of a software. EDAM ontology [135] can be used to annotate input data type, input data format, output data type, output data format, and operation of a tool in the workflow. Software Ontology (SWO) extended EDAM ontology and linked data types and formats to a taxonomy of software [136]. WICUS ontology describes underlying hardware and computational infrastructure [137]. To implement semantic annotation of workflows, several semantic workflow development platforms were created by researchers, such as jORCA/Magallanes [138], jABC/PROPHETS [139,140], WINGS [141], and APE [142]. Such platforms can find workflows automatically based on the annotation; the technique is also called automated workflow composition. Unlike other automated workflow composition approaches such as searching through a workflow repository like myExperiment [143], which may have an 80% probability to return a decayed workflow [144], automated workflow composition using annotations from an ontological-based controlled vocabulary can discover and create new usable workflows. Automated workflow composition with controlled vocabulary was implemented to several types of data, including proteomics, genomics, and geographical data [131,145,146,147]; it can also be used for metabolomics data. Therefore, semantic annotation of computational workflow allows the recreation of new and similar in the case that the workflow in the original publication is obsolete, thus addressing the issue of workflow decay, and multiple semantic workflow development platforms were created to facilitate the process.

### 4.6. Use Workflow Automation or Literate Programming

Recently, Heil et al. suggested that reproducibility is not only about enabling a second researcher to regenerate the results but also related to how fast or easy a second researcher can get the result [45]. A study with results that can only be regenerated by consulting the original author intensively will be less reproducible than a study with results that can be recreated using one simple command line. In terms of reducing the effort of reproducing the original results, literate programming and workflow automation are two feasible techniques. Literate programming combines a narrative description of the research with code. A document including code, narratives, and any outputs (e.g., tables, figures) of the code will be produced after execution. To some extent, literate programming helps readers understand exactly how a particular result was obtained. By reducing difficulties of understanding among researchers, literate programming can facilitate greater trust in computational findings [148]. Two popular tools for literate programming are Jupyter Notebook [149] and knitr [150,151]. Jupyter Notebook is an open-source web application. With Jupyter Notebook, researchers can create and share documents containing live code, mathematical formulas and equations, and visualizations. Jupyter Notebook supports several programming languages, including Python [152], R [153], and Shell [154]. Knitr is similar to Jupyter Notebook but written in R programming language, which also gained considerable popularity as a literate programming tool. On the other hand, workflow automation also benefits reproducibility, which connects all processes in the workflow with software or code. Workflow automation reduces hands-on steps, making it faster and easier to regenerate the initial results, as well as reducing human error. Galaxy and Nextflow are two workflow automation platforms that were used in metabolomics field. Galaxy is a web-based scientific workflow automation platform that was widely used in the bioinformatics area. Galaxy has a graphical user interface, making it easier for research scientists who do not have computer programming experience [155]. Nextflow is a workflow automation platform written with Groovy programming language [120]. Nextflow also supports several workflow schedulers, making it suitable for high-performance computing and large-scale data analysis. However, Nextflow is not friendly to a scientist with few programming experiences. In summary, it is recommended for metabolomics researchers to provide literate programming or automated workflow to enhance reproducibility.

## 5. Conclusions

Metabolomics is an emerging field and was widely used in clinical studies [153,156]. However, efforts towards improving the reproducibility of metabolomics data analysis pipelines are still in their infancy. There was a clinical need for making clinical metabolomics research reproducible. In this paper, we proposed a checklist by summarizing techniques and tools that can enhance metabolomics research reproducibility. Unlike existing checklists for improving reproducibility in other fields [26,27], which contain tens of items, our proposed checklist only has eight items, making it concise and easy to follow. Each item was explained in detail; tools corresponding to each item were also recommended. However, the effectiveness of the checklist may still need to be tested and quantified in the future. In conclusion, the proposed checklist may benefit authors, reviewers, editors, and readers in the clinical metabolomics field by making studies more robust and reliable. More efforts are needed from the scientific community to ensure reproducible metabolomics research and to make metabolomics research results more reliable and trustworthy before being applied in real clinical settings.

## Figures and Tables

**Table 1:** Ontologies that can provide semantic annotation for computational workflows.

Ontology Name	Owl File
Research Object Ontology	http://purl.org/wf4ever/ro#, accessed on 29 November 2021
Workflow Description Ontology	http://purl.org/wf4ever/wfdesc#, accessed on 29 November 2021
Workflow Provenance Ontology	http://purl.org/wf4ever/wfprov#, accessed on 29 November 2021
Research Object Evolution Ontology	http://purl.org/wf4ever/roevo#, accessed on 29 November 2021
OntoSoft Ontology	http://ontosoft-earthcube.github.io/ontosoft/ontosoft%20ontology/v1.0.1/doc/ontosoft-v1.0.1.owl, accessed on 29 November 2021
Software Description Ontology	https://w3id.org/okn/o/sd, accessed on 29 November 2021
DOAP Ontology	http://usefulinc.com/ns/doap, accessed on 29 November 2021
EDAM Ontology	http://edamontology.org/EDAM.owl, accessed on 29 November 2021
Software Ontology	http://www.ebi.ac.uk/swo/swo.owl, accessed on 29 November 2021
WICUS Ontology	http://vocab.linkeddata.es/wicus/hwspecs/hwspecs.owl, accessed on 29 November 2021

## References

[1] Johnson C.H., Ivanisevic J., Siuzdak G. (2016). Metabolomics: Beyond biomarkers and towards mechanisms. Nat. Rev. Mol. Cell Biol..

[2] Schmidt D.R., Patel R., Kirsch D.G., Lewis C.A., Vander Heiden M.G., Locasale J.W. (2021). Metabolomics in cancer research and emerging applications in clinical oncology. CA Cancer J. Clin..

[3] Balashova E.E., Maslov D.L., Lokhov P.G. (2018). A Metabolomics Approach to Pharmacotherapy Personalization. J. Pers. Med..

[4] Trivedi D.K., Hollywood K.A., Goodacre R. (2017). Metabolomics for the masses: The future of metabolomics in a personalized world. Eur. J. Mol. Clin. Med..

[5] Helland T., Hagen K.B., Haugstøyl M.E., Kvaløy J.T., Lunde S., Lode K., Lind R.A., Gripsrud B.H., Jonsdottir K., Gjerde J. (2019). Drug monitoring of tamoxifen metabolites predicts vaginal dryness and verifies a low discontinuation rate from the Norwegian Prescription Database. Breast Cancer Res. Treat..

[6] Pevsner P.H., Melamed J., Remsen T., Kogos A., Francois F., Kessler P., Stern A., Anand S. (2009). Mass spectrometry MALDI imaging of colon cancer biomarkers: A new diagnostic paradigm. Biomark. Med..

[7] Rebholz C.M., Yu B., Zheng Z., Chang P., Tin A., Köttgen A., Wagenknecht L.E., Coresh J., Boerwinkle E., Selvin E. (2018). Serum metabolomic profile of incident diabetes. Diabetologia.

[8] Luo P., Yin P., Hua R., Tan Y., Li Z., Qiu G., Yin Z., Xie X., Wang X., Chen W. (2018). A Large-scale, multicenter serum metabolite biomarker identification study for the early detection of hepatocellular carcinoma. Hepatology.

[9] Zhang X.-W., Li Q.-H., Xu Z.-D., Dou J.-J. (2020). Mass spectrometry-based metabolomics in health and medical science: A systematic review. RSC Adv..

[10] O’Gorman A., Brennan L. (2017). The role of metabolomics in determination of new dietary biomarkers. Proc. Nutr. Soc..

[11] Giovannini G., Haick H., Garoli D. (2021). Detecting COVID-19 from Breath: A Game Changer for a Big Challenge. ACS Sens..

[12] Fraser D.D., Slessarev M., Martin C.M., Daley M., Patel M.A., Miller M.R., Patterson E.K., O’Gorman D.B., Gill S.E., Wishart D.S. (2020). Metabolomics Profiling of Critically Ill Coronavirus Disease 2019 Patients: Identification of Diagnostic and Prognostic Biomarkers. Crit. Care Explor..

[13] Barberis E., Timo S., Amede E., Vanella V.V., Puricelli C., Cappellano G., Raineri D., Cittone M.G., Rizzi E., Pedrinelli A.R. (2020). Large-Scale Plasma Analysis Revealed New Mechanisms and Molecules Associated with the Host Response to SARS-CoV-2. Int. J. Mol. Sci..

[14] Hasan M.R., Suleiman M., Pérez-López A. (2021). Metabolomics in the Diagnosis and Prognosis of COVID. Front. Genet..

[15] Franceschi P., Mylonas R., Shahaf N., Scholz M., Arapitsas P., Masuero D., Weingart G., Carlin S., Vrhovsek U., Mattivi F. (2014). MetaDB a Data Processing Workflow in Untargeted MS-Based Metabolomics Experiments. Front. Bioeng. Biotechnol..

[16] Yu M., Dolios G., Petrick L. (2021). Reproducible Untargeted Metabolomics Data Analysis Workflow for Exhaustive MS/MS Annotation. Anal. Chem..

[17] Siskos A.P., Jain P., Römisch-Margl W., Bennett M., Achaintre D., Asad Y., Marney L., Richardson L., Koulman A., Griffin J.L. (2017). Interlaboratory Reproducibility of a Targeted Metabolomics Platform for Analysis of Human Serum and Plasma. Anal. Chem..

[18] Tebani A., Abily-Donval L., Afonso C., Marret S., Bekri S. (2016). Clinical Metabolomics: The New Metabolic Window for Inborn Errors of Metabolism Investigations in the Post-Genomic Era. Int. J. Mol. Sci..

[19] Lin Y., Caldwell G.W., Li Y., Lang W., Masucci J. (2020). Inter-laboratory reproducibility of an untargeted metabolomics GC–MS assay for analysis of human plasma. Sci. Rep..

[20] United States, Food and Drug Administration, Office of Combination Products (2006). Guidance for Industry and FDA Staff.

[21] Shao J., Chow S.-C. (2002). Reproducibility probability in clinical trials. Stat. Med..

[22] FDA (2021). Advancing Regulatory Science at FDA: Focus Areas of Regulatory Science (FARS).

[23] Han S., Olonisakin T.F., Pribis J.P., Zupetic J., Yoon J.H., Holleran K., Jeong K., Shaikh N., Rubio D.M., Lee J. (2017). A checklist is associated with increased quality of reporting preclinical biomedical research: A systematic review. PLoS ONE.

[24] Hales B., Terblanche M., Fowler R., Sibbald W. (2007). Development of medical checklists for improved quality of patient care. Int. J. Qual. Health Care.

[25] Chaparro A., Keebler J., Lazzara E.H., Diamond A. (2019). Checklists: A Review of Their Origins, Benefits, and Current Uses as a Cognitive Aid in Medicine. Ergon. Des. Q. Hum. Factors Appl..

[26] Schwendicke F., Singh T., Lee J.-H., Gaudin R., Chaurasia A., Wiegand T., Uribe S., Krois J. (2021). Artificial intelligence in dental research: Checklist for authors, reviewers, readers. J. Dent..

[27] Feng X., Park D.S., Walker C., Peterson A.T., Merow C., Papeş M. (2019). A checklist for maximizing reproducibility of ecological niche models. Nat. Ecol. Evol..

[28] Fiehn O., Robertson D., Griffin J., Van Der Werf M., Nikolau B., Morrison N., Sumner L.W., Goodacre R., Hardy N.W., Taylor C. (2007). The metabolomics standards initiative (MSI). Metabolomics.

[29] Salek R.M., Steinbeck C., Viant M.R., Goodacre R., Dunn W.B. (2013). The role of reporting standards for metabolite annotation and identification in metabolomic studies. GigaScience.

[30] Sumner L.W., Amberg A., Barrett D., Beale M.H., Beger R., Daykin C.A., Fan T.W.-M., Fiehn O., Goodacre R., Griffin J.L. (2007). Proposed minimum reporting standards for chemical analysis. Metabolomics.

[31] Goodacre R., Broadhurst D., Smilde A.K., Kristal B.S., Baker J.D., Beger R., Bessant C., Connor S., Capuani G., Craig A. (2007). Proposed minimum reporting standards for data analysis in metabolomics. Metabolomics.

[32] Morrison N., Bearden D., Bundy J.G., Collette T., Currie F., Davey M.P., Watson-Haigh N., Hancock D., Jones O., Rochfort S. (2007). Standard reporting requirements for biological samples in metabolomics experiments: Environmental context. Metabolomics.

[33] Griffin J.L., Nicholls A.W., Daykin C.A., Heald S., Keun H.C., Schuppe-Koistinen I., Griffiths J.R., Cheng L.L., Rocca-Serra P., Rubtsov D.V. (2007). Standard reporting requirements for biological samples in metabolomics experiments: Mammalian/in vivo experiments. Metabolomics.

[34] van der Werf M.J., Takors R., Smedsgaard J., Nielsen J., Ferenci T., Portais J.C., Wittmann C., Hooks M., Tomassini A., Oldiges M. (2007). Standard reporting requirements for biological samples in metabolomics experiments: Microbial and in vitro biology experiments. Metabolomics.

[35] Fiehn O., Sumner L.W., Rhee S.Y., Ward J., Dickerson J., Lange B.M., Lane G., Roessner U., Last R., Nikolau B. (2007). Minimum reporting standards for plant biology context information in metabolomic studies. Metabolomics.

[36] Rubtsov D.V., Jenkins H., Ludwig C., Easton J., Viant M.R., Günther U., Griffin J.L., Hardy N. (2007). Proposed reporting requirements for the description of NMR-based metabolomics experiments. Metabolomics.

[37] Snyder M., Mias G., Stanberry L., Kolker E. (2013). Metadata Checklist for the Integrated Personal Omics Study: Proteomics and Metabolomics Experiments. Big Data.

[38] Long N.P., Nghi T.D., Kang Y.P., Anh N.H., Kim H.M., Park S.K., Kwon S.W. (2020). Toward a Standardized Strategy of Clinical Metabolomics for the Advancement of Precision Medicine. Metabolites.

[39] Considine E.C., Salek R.M. (2019). A Tool to Encourage Minimum Reporting Guideline Uptake for Data Analysis in Metabolomics. Metabolites.

[40] Viant M.R., Ebbels T.M.D., Beger R.D., Ekman D.R., Epps D.J.T., Kamp H., Leonards P.E.G., Loizou G.D., Macrae J.I., Van Ravenzwaay B. (2019). Use cases, best practice and reporting standards for metabolomics in regulatory toxicology. Nat. Commun..

[41] Considine E.C., Thomas G., Boulesteix A.L., Khashan A.S., Kenny L.C. (2017). Critical review of reporting of the data analysis step in metabolomics. Metabolomics.

[42] Spicer R.A., Salek R., Steinbeck C. (2017). A decade after the metabolomics standards initiative it’s time for a revision. Sci. Data.

[43] Nasir L. (2010). The Checklist Manifesto: How to Get Things Right. Lond. J. Prim. Care.

[44] Spicer R.A., Salek R., Steinbeck C. (2017). Compliance with minimum information guidelines in public metabolomics repositories. Sci. Data.

[45] Heil B.J., Hoffman M.M., Markowetz F., Lee S.-I., Greene C.S., Hicks S.C. (2021). Reproducibility standards for machine learning in the life sciences. Nat. Methods.

[46] Cambiaghi A., Ferrario M., Masseroli M. (2016). Analysis of metabolomic data: Tools, current strategies and future challenges for omics data integration. Briefings Bioinform..

[47] Nalbantoglu S. (2019). Metabolomics: Basic Principles and Strategies. Mol. Med..

[48] Lee J.-E., Kim Y.-Y. (2017). Impact of Preanalytical Variations in Blood-Derived Biospecimens on Omics Studies: Toward Precision Biobanking?. OMICS.

[49] Kirwan J.A., Brennan L., Broadhurst D., Fiehn O., Cascante M., Dunn W.B., Schmidt M.A., Velagapudi V. (2018). Preanalytical Processing and Biobanking Procedures of Biological Samples for Metabolomics Research: A White Paper, Community Perspective (for “Precision Medicine and Pharmacometabolomics Task Group”—The Metabolomics Society Initiative). Clin. Chem..

[50] Bi H., Guo Z., Jia X., Liu H., Ma L., Xue L. (2020). The key points in the pre-analytical procedures of blood and urine samples in metabolomics studies. Metabolomics.

[51] Biais B., Bernillon S., Deborde C., Cabasson C., Rolin M., Tadmor Y., Burger J., Schaffer A.A., Moing A. (2011). Precautions for Harvest, Sampling, Storage, and Transport of Crop Plant Metabolomics Samples. Advanced Structural Safety Studies.

[52] Smith L., Villaret-Cazadamont J., Claus S.P., Canlet C., Guillou H., Cabaton N.J., Ellero-Simatos S. (2020). Important Considerations for Sample Collection in Metabolomics Studies with a Special Focus on Applications to Liver Functions. Metabolites.

[53] Nussbeck S.Y., Skrowny D., O’Donoghue S., Schulze T.G., Helbing K. (2014). How to Design Biospecimen Identifiers and Integrate Relevant Functionalities into Your Biospecimen Management System. Biopreservation Biobanking.

[54] Cooper D.R., Grabowski M., Zimmerman M.D., Porebski P.J., Shabalin I.G., Woinska M., Domagalski M.J., Zheng H., Sroka P., Cymborowski M. (2021). State-of-the-Art Data Management: Improving the Reproducibility, Consistency, and Traceability of Structural Biology and in Vitro Biochemical Experiments. Methods Mol. Biol..

[55] Macneil R. (2011). The benefits of integrated systems for managing both samples and experimental data: An opportunity for labs in universities and government research institutions to lead the way. Autom. Exp..

[56] Rasmussen L., Wilhelmsen M., Christensen I.J., Andersen J., Jørgensen L.N., Rasmussen M., Hendel J.W., Madsen M.R., Vilandt J., Hillig T. (2016). Protocol Outlines for Parts 1 and 2 of the Prospective Endoscopy III Study for the Early Detection of Colorectal Cancer: Validation of a Concept Based on Blood Biomarkers. JMIR Res. Protoc..

[57] Faraji M., Yamini Y., Gholami M. (2019). Recent Advances and Trends in Applications of Solid-Phase Extraction Techniques in Food and Environmental Analysis. Chromatographia.

[58] Sana T., Fischer S., Clara S. (2007). Maximizing Metabolite Extraction for Comprehensive Metabolomics Studies of Erythrocytes. Agil. Technol..

[59] Gong Z.-G., Hu J., Wu X., Xu Y.-J. (2016). The Recent Developments in Sample Preparation for Mass Spectrometry-Based Metabolomics. Crit. Rev. Anal. Chem..

[60] Andrade-Eiroa A., Canle M., Leroy-Cancellieri V., Cerdà V. (2016). Solid-phase extraction of organic compounds: A critical review (Part I). TrAC Trends Anal. Chem..

[61] Chen X., Xu F., Wang Y., Pan Y., Lu D., Wang P., Ying K., Chen E., Zhang W. (2007). A study of the volatile organic compounds exhaled by lung cancer cells in vitro for breath diagnosis. Cancer.

[62] Danaceau J., Haynes K., Chambers E. A Comprehensive Comparison of Solid Phase Extraction (SPE) vs. Solid Liquid Extraction (SLE) vs. Liquid Liquid Extraction (LLE) Sample Prep Techniques in Bioanalysis and Forensic Toxicology Analyses. https://www.waters.com/nextgen/xg/en/library/application-notes/2017/solid-phase-extraction-vs-solid-liquid-extraction-vs-liquid-liquid-extraction.html.

[63] Liu R., Chou J., Hou S., Liu X., Yu J., Zhao X., Li Y., Liu L., Sun C. (2018). Evaluation of two-step liquid-liquid extraction protocol for untargeted metabolic profiling of serum samples to achieve broader metabolome coverage by UPLC-Q-TOF-MS. Anal. Chim. Acta.

[64] Adusumilli R., Mallick P. (2017). Data Conversion with ProteoWizard msConvert. Proteomics.

[65] Emwas A.-H., Roy R., McKay R.T., Tenori L., Saccenti E., Gowda G.A.N., Raftery D., AlAhmari F., Jaremko L., Jaremko M. (2019). NMR Spectroscopy for Metabolomics Research. Metabolites.

[66] Urban P.L. (2016). Quantitative mass spectrometry: An overview. Philos. Trans. R. Soc. A Math. Phys. Eng. Sci..

[67] Cajka T., Fiehn O. (2016). Toward Merging Untargeted and Targeted Methods in Mass Spectrometry-Based Metabolomics and Lipidomics. Anal. Chem..

[68] Wishart D.S. (2009). Computational strategies for metabolite identification in metabolomics. Bioanalysis.

[69] Beale D.J., Pinu F.R., Kouremenos K.A., Poojary M.M., Narayana V.K., Boughton B.A., Kanojia K., Dayalan S., Jones O.A.H., Dias D.A. (2018). Review of recent developments in GC–MS approaches to metabolomics-based research. Metabolomics.

[70] Misra B.B., Olivier M. (2020). High Resolution GC-Orbitrap-MS Metabolomics Using Both Electron Ionization and Chemical Ionization for Analysis of Human Plasma. J. Proteome Res..

[71] Stein S.E. (1999). An integrated method for spectrum extraction and compound identification from gas chromatography/mass spectrometry data. J. Am. Soc. Mass Spectrom..

[72] Pitt J.J. (2009). Principles and Applications of Liquid Chromatography-Mass Spectrometry in Clinical Biochemistry. Clin. Biochem. Rev..

[73] Malviya R., Bansal V., Pal O.P., Sharma P.K. (2010). High performance liquid chromatography: A short review. J. Glob. Pharma Technol..

[74] Xiao J.F., Zhou B., Ressom H.W. (2012). Metabolite identification and quantitation in LC-MS/MS-based metabolomics. TrAC Trends Anal. Chem..

[75] Varghese R.S., Zhou B., Ranjbar M.R.N., Zhao Y., Ressom H.W. (2012). Ion annotation-assisted analysis of LC-MS based metabolomic experiment. Proteome Sci..

[76] Hillenkamp F., Karas M., Beavis R.C., Chait B.T. (1991). Matrix-Assisted Laser Desorption/Ionization Mass Spectrometry of Biopolymers. Anal. Chem..

[77] Hoegen B., Zammit A., Gerritsen A., Engelke U.F.H., Castelein S., van de Vorst M., Kluijtmans L.A.J., Huigen M.C.D.G., Wevers R.A., van Gool A.J. (2021). Metabolomics-Based Screening of Inborn Errors of Metabolism: Enhancing Clinical Application with a Robust Computational Pipeline. Metabolites.

[78] Pluskal T., Castillo S., Villar-Briones A., Orešič M. (2010). MZmine 2: Modular framework for processing, visualizing, and analyzing mass spectrometry-based molecular profile data. BMC Bioinform..

[79] Smith C.A., Want E.J., O’Maille G., Abagyan R., Siuzdak G. (2006). XCMS: Processing Mass Spectrometry Data for Metabolite Profiling Using Nonlinear Peak Alignment, Matching, and Identification. Anal. Chem..

[80] Pang Z.Q., Chong J., Zhou G.Y., de Lima Morais D.A., Chang L., Barrette M., Gauthier C., Jacques P.-É., Li S.Z., Xia J.G. (2021). MetaboAnalyst 5.0: Narrowing the gap between raw spectra and functional insights. Nucleic Acids Res..

[81] Röst H., Sachsenberg T., Aiche S., Bielow C., Weisser H., Aicheler F., Andreotti S., Ehrlich H.-C., Gutenbrunner P., Kenar E. (2016). OpenMS: A flexible open-source software platform for mass spectrometry data analysis. Nat. Methods.

[82] Tsugawa H., Cajka T., Kind T., Ma Y., Higgins B., Ikeda K., Kanazawa M., VanderGheynst J., Fiehn O., Arita M. (2015). MS-DIAL: Data-independent MS/MS deconvolution for comprehensive metabolome analysis. Nat. Methods.

[83] Teruya T., Chen Y.-J., Kondoh H., Fukuji Y., Yanagida M. (2021). Whole-blood metabolomics of dementia patients reveal classes of disease-linked metabolites. Proc. Natl. Acad. Sci. USA.

[84] Altadill T., Campoy I., Lanau L., Gill K., Rigau M., Gil-Moreno A., Reventos J., Byers S., Colas E., Cheema A.K. (2016). Enabling Metabolomics Based Biomarker Discovery Studies Using Molecular Phenotyping of Exosome-Like Vesicles. PLoS ONE.

[85] Liu X., Jin F., Wang C., Zhao S., Han S., Jiang P., Cui C. (2021). Targeted metabolomics analysis of serum amino acid profiles in patients with Moyamoya disease. Amino Acids.

[86] McCall L.-I., Tripathi A., Vargas F., Knight R., Dorrestein P.C., Siqueira-Neto J.L. (2018). Experimental Chagas disease-induced perturbations of the fecal microbiome and metabolome. PLoS Negl. Trop. Dis..

[87] Klont F., Kremer D., Neto A.W.G., Berger S.P., Touw D.J., Hak E., Bonner R., Bakker S.J., Hopfgartner G. (2021). Metabolomics data complemented drug use information in epidemiological databases: Pilot study of potential kidney donors. J. Clin. Epidemiol..

[88] Alzubaidi L., Zhang J., Humaidi A.J., Al-Dujaili A., Duan Y., Al-Shamma O., Santamaría J., Fadhel M.A., Al-Amidie M., Farhan L. (2021). Review of deep learning: Concepts, CNN architectures, challenges, applications, future directions. J. Big Data.

[89] Kantz E.D., Tiwari S., Watrous J.D., Cheng S., Jain M. (2019). Deep Neural Networks for Classification of LC-MS Spectral Peaks. Anal. Chem..

[90] Melnikov A.D., Tsentalovich Y.P., Yanshole V.V. (2020). Deep Learning for the Precise Peak Detection in High-Resolution LC–MS Data. Anal. Chem..

[91] Zohora F.T., Rahman M.Z., Tran N.H., Xin L., Shan B., Li M. (2019). DeepIso: A Deep Learning Model for Peptide Feature Detection from LC-MS map. Sci. Rep..

[92] Gloaguen Y., Kirwan J., Beule D. (2020). Deep Learning assisted Peak Curation for large scale LC-MS Metabolomic. bioRxiv.

[93] Dunn W.B., Broadhurst D., Begley P., Zelena E., Francis-McIntyre S., Anderson N., Brown M., Knowles J.D., Halsall A., Haselden J.N. (2011). Procedures for large-scale metabolic profiling of serum and plasma using gas chromatography and liquid chromatography coupled to mass spectrometry. Nat. Protoc..

[94] Misra B.B. (2020). Data normalization strategies in metabolomics: Current challenges, approaches, and tools. Eur. J. Mass Spectrom..

[95] Reinhold D., Pielke-Lombardo H., Jacobson S., Ghosh D., Kechris K. (2019). Pre-analytic Considerations for Mass Spectrometry-Based Untargeted Metabolomics Data. High-Throughput Metab..

[96] Schrimpe-Rutledge A.C., Codreanu S.G., Sherrod S.D., McLean J.A. (2016). Untargeted Metabolomics Strategies—Challenges and Emerging Directions. J. Am. Soc. Mass Spectrom..

[97] Liu Q., Walker D., Uppal K., Liu Z., Ma C., Tran V., Li S., Jones D.P., Yu T. (2020). Addressing the batch effect issue for LC/MS metabolomics data in data preprocessing. Sci. Rep..

[98] Bartel J., Krumsiek J., Theis F.J. (2013). Statistical methods for the analysis of high-throughput metabolomics data. Comput. Struct. Biotechnol. J..

[99] De Livera A.M., Olshansky M., Speed T.P. (2013). Statistical Analysis of Metabolomics Data. Methods Mol. Biol. Clifton NJ.

[100] Liebal U.W., Phan A.N.T., Sudhakar M., Raman K., Blank L.M. (2020). Machine Learning Applications for Mass Spectrometry-Based Metabolomics. Metabolites.

[101] Majumder E.L.-W., Billings E.M., Benton H.P., Martin R.L., Palermo A., Guijas C., Rinschen M.M., Domingo-Almenara X., Montenegro-Burke J.R., Tagtow B.A. (2021). Cognitive analysis of metabolomics data for systems biology. Nat. Protoc..

[102] Rocca-Serra P., Salek R.M., Arita M., Correa E., Dayalan S., Gonzalez-Beltran A., Ebbels T., Goodacre R., Hastings J., Haug K. (2016). Data standards can boost metabolomics research, and if there is a will, there is a way. Metabolomics.

[103] Haug K., Cochrane K., Nainala V.C., Williams M., Chang J., Jayaseelan K.V., O’Donovan C. (2019). MetaboLights: A resource evolving in response to the needs of its scientific community. Nucleic Acids Res..

[104] Sud M., Fahy E., Cotter D., Azam K., Vadivelu I., Burant C., Edison A., Fiehn O., Higashi R., Nair K.S. (2016). Metabolomics Workbench: An international repository for metabolomics data and metadata, metabolite standards, protocols, tutorials and training, and analysis tools. Nucleic Acids Res..

[105] Neef S.K., Janssen N., Winter S., Wallisch S.K., Hofmann U., Dahlke M.H., Schwab M., Mürdter T.E., Haag M. (2020). Metabolic Drug Response Phenotyping in Colorectal Cancer Organoids by LC-QTOF-MS. Metabolites.

[106] Wu M., Chen W., Miao M., Jin Q., Zhang S., Bai M., Fan J., Zhang Y., Zhang A., Jia Z. (2021). Anti-anemia drug FG4592 retards the AKI-to-CKD transition by improving vascular regeneration and antioxidative capability. Clin. Sci..

[107] Getting the Facts Right|UNECE. https://unece.org/getting-facts-right.

[108] STAR Methods: Cell Press. https://www.cell.com/star-methods.

[109] Pavlovich M.J., Buttery S. (2021). How peer review and publication can make a good protocol even better. STAR Protoc..

[110] Li S., Sullivan N.L., Rouphael N., Yu T., Banton S., Maddur M.S., McCausland M., Chiu C., Canniff J., Dubey S. (2017). Metabolic Phenotypes of Response to Vaccination in Humans. Cell.

[111] Xu F., Song C., Liu W., Chen G. (2021). Protocol for intracellular and extracellular metabolite detection in human embryonic stem cells. STAR Protoc..

[112] Meng Y., Ren Z., Xu F., Zhou X., Song C., Wang V.Y.-F., Liu W., Lu L., Thomson J.A., Chen G. (2018). Nicotinamide Promotes Cell Survival and Differentiation as Kinase Inhibitor in Human Pluripotent Stem Cells. Stem Cell Rep..

[113] Song C., Xu F., Ren Z., Zhang Y., Meng Y., Yang Y., Lingadahalli S., Cheung E., Li G., Liu W. (2019). Elevated Exogenous Pyruvate Potentiates Mesodermal Differentiation through Metabolic Modulation and AMPK/mTOR Pathway in Human Embryonic Stem Cells. Stem Cell Rep..

[114] Yang Y., Ren Z., Xu F., Meng Y., Zhang Y., Ai N., Long Y., Fok H.I., Deng C., Zhao X. (2019). Endogenous IGF Signaling Directs Heterogeneous Mesoderm Differentiation in Human Embryonic Stem Cells. Cell Rep..

[115] Blischak J.D., Davenport E.R., Wilson G. (2016). A Quick Introduction to Version Control with Git and GitHub. PLoS Comput. Biol..

[116] Alvarez-Mulett S., Buyukozkan M., Racanelli A.C., Schmidt F., Batra R., Hoffman K.L., Sarwath H., Engelke R., Gomez-Escobar L., Simmons W. (2021). Integrative Metabolomic and Proteomic Signatures Define Clinical Outcomes in Severe COVID-19. medRxiv.

[117] Nederbragt A.J. (2014). On the middle ground between open source and commercial software—The case of the Newbler program. Genome Biol..

[118] Ince D.C., Hatton L., Graham-Cumming J. (2012). The case for open computer programs. Nat. Cell Biol..

[119] Schloss P.D. (2018). Identifying and Overcoming Threats to Reproducibility, Replicability, Robustness, and Generalizability in Microbiome Research. mBio.

[120] Di Tommaso P., Chatzou M., Floden E.W., Barja P.P., Palumbo E., Notredame C. (2017). Nextflow enables reproducible computational workflows. Nat. Biotechnol..

[121] Wiebels K., Moreau D. (2021). Leveraging Containers for Reproducible Psychological Research. Adv. Methods Pract. Psychol. Sci..

[122] Viereck M. (2019). x11docker: Run GUI applications in Docker containers. J. Open Source Softw..

[123] Hung L.-H., Kristiyanto D., Lee S.B., Yeung K.Y. (2016). GUIdock: Using Docker Containers with a Common Graphics User Interface to Address the Reproducibility of Research. PLoS ONE.

[124] Senington R., Pataki B., Wang X.V. (2018). Using docker for factory system software management: Experience report. Procedia CIRP.

[125] Merkel D. (2014). Docker: Lightweight Linux containers for consistent development and deployment. Linux J..

[126] Kurtzer G.M., Sochat V., Bauer M.W. (2017). Singularity: Scientific containers for mobility of compute. PLoS ONE.

[127] Arango C., Dernat R., Sanabria J. (2019). Performance evaluation of container-based virtualization for high performance computing environments. Revista UIS Ingenierías.

[128] Documentation—Oracle VM VirtualBox. https://www.virtualbox.org/wiki/Documentation.

[129] Lamprecht A.-L., Palmblad M., Ison J., Schwämmle V., Al Manir M.S., Altintas I., Baker C.J.O., Amor A.B.H., Capella-Gutierrez S., Charonyktakis P. (2021). Perspectives on automated composition of workflows in the life sciences. F1000Research.

[130] Belhajjame K., Zhao J., Garijo D., Gamble M., Hettne K., Palma R., Mina E., Corcho O., Gómez-Pérez J.M., Bechhofer S. (2015). Using a suite of ontologies for preserving workflow-centric research objects. J. Web Semant..

[131] Palmblad M., Lamprecht A.-L., Ison J., Schwämmle V. (2019). Automated workflow composition in mass spectrometry-based proteomics. Bioinform..

[132] Gil Y., Ratnakar V., Garijo D. OntoSoft. Proceedings of the 8th International Conference on Knowledge Capture.

[133] Carvalho L.A.M.C., Garijo D., Medeiros C.B., Gil Y. Semantic Software Metadata for Workflow Exploration and Evolution. Proceedings of the 2018 IEEE 14th International Conference on e-Science (e-Science).

[134] Garijo D., Osorio M., Khider D., Ratnakar V., Gil Y. OKG-Soft: An Open Knowledge Graph with Machine Readable Scientific Software Metadata. Proceedings of the 2019 15th International Conference on eScience (eScience).

[135] Ison J., Kalaš M., Jonassen I., Bolser D., Uludag M., McWilliam H., Malone J.R., López R., Pettifer S., Rice P. (2013). EDAM: An ontology of bioinformatics operations, types of data and identifiers, topics and formats. Bioinformatics.

[136] Malone J., Brown A., Lister A.L., Ison J., Hull D., Parkinson H., Stevens R. (2014). The Software Ontology (SWO): A resource for reproducibility in biomedical data analysis, curation and digital preservation. J. Biomed. Semant..

[137] Santana-Perez I., Hernandez M.D.L.S.P. (2015). Towards Reproducibility in Scientific Workflows: An Infrastructure-Based Approach. Sci. Program..

[138] Karlsson J., Martín-Requena V., Ríos J., Trelles O. (2010). Workflow Composition and Enactment Using jORCA. Leveraging Applications of Formal Methods, Verification, and Validation.

[139] Lamprecht A.-L., Naujokat S., Margaria T., Steffen B. Synthesis-Based Loose Programming. Proceedings of the 2010 Seventh International Conference on the Quality of Information and Communications Technology.

[140] Naujokat S., Lamprecht A.-L., Steffen B. (2012). Loose Programming with PROPHETS. Fundamental Approaches to Software Engineering.

[141] Gil Y., Ratnakar V., Kim J., Gonzalez-Calero P.A., Groth P., Moody J., Deelman E. (2010). Wings: Intelligent Workflow-Based Design of Computational Experiments. IEEE Intell. Syst..

[142] Kasalica V., Lamprecht A.-L. (2020). APE: A Command-Line Tool and API for Automated Workflow Composition. Security and Trust Management.

[143] Goble C.A., Bhagat J., Aleksejevs S., Cruickshank D., Michaelides D., Newman D., Borkum M., Bechhofer S., Roos M., Li P. (2010). myExperiment: A repository and social network for the sharing of bioinformatics workflows. Nucleic Acids Res..

[144] Zhao J., Gomez-Perez J.M., Belhajjame K., Klyne G., Garcia-Cuesta E., Garrido A., Hettne K., Roos M., De Roure D., Goble C. Why workflows break Understanding and combating decay in Taverna workflows. Proceedings of the 2012 IEEE 8th International Conference on E-Science.

[145] Kasalica V., Schwämmle V., Palmblad M., Ison J., Lamprecht A.-L. (2021). APE in the Wild: Automated Exploration of Proteomics Workflows in the bio.tools Registry. J. Proteome Res..

[146] Kasalica V., Lamprecht A.-L. Automated composition of scientific workflows: A case study on geographic data manipulation. Proceedings of the 2018 IEEE 14th International Conference on e-Science (e-Science).

[147] Zheng C.L., Ratnakar V., Gil Y., McWeeney S.K. (2015). Use of semantic workflows to enhance transparency and reproducibility in clinical omics. Genome Med..

[148] Piccolo S.R., Frampton M.B. (2016). Tools and techniques for computational reproducibility. GigaScience.

[149] Kluyver T., Ragan-Kelley B., Pérez F., Granger B., Bussonnier M., Frederic J., Kelley K., Hamrick J., Grout J., Corlay S. (2016). Jupyter Notebooks—A Publishing Format for Reproducible Computational Workflows.

[150] Xie Y. (2018). knitr: A Comprehensive Tool for Reproducible Research in R. Implementing Reproducible Research.

[151] Xie Y. knitr. https://yihui.org/knitr/.

[152] van Rossum G., Drake F.L. (2009). Python 3 Reference Manual.

[153] R Core Team R: A Language and Environment for Statistical Computing. https://www.R-project.org/.

[154] Bash–GNU Project–Free Software Foundation. https://www.gnu.org/software/bash/.

[155] Afgan E., Baker D., Batut B., van den Beek M., Bouvier D., Čech M., Chilton J., Clements D., Coraor N., Grüning B.A. (2018). The Galaxy platform for accessible, reproducible and collaborative biomedical analyses: 2018 update. Nucleic Acids Res..

[156] Ulmer C.Z., Maus A., Hines J., Singh R. (2021). Challenges in Translating Clinical Metabolomics Data Sets from the Bench to the Bedside. Clin. Chem..

